# Chemical Diversity and Antimicrobial Potential of Cultivable Fungi from Deep-Sea Sediments of the Gulf of Mexico

**DOI:** 10.3390/molecules26237328

**Published:** 2021-12-02

**Authors:** Rodrigo Villanueva-Silva, Patricia Velez, Meritxell Riquelme, Carlos A. Fajardo-Hernández, Anahí Martínez-Cárdenas, Alejandra Arista-Romero, Baojie Wan, Rui Ma, Mallique Qader, Scott G. Franzblau, Mario Figueroa

**Affiliations:** 1 Facultad de Química, Universidad Nacional Autónoma de México, Ciudad de México 04510, Mexico; code_rodvil@comunidad.unam.mx (R.V.-S.); cfajardo@quimica.unam.mx (C.A.F.-H.); amartinez@quimica.unam.mx (A.M.-C.); aleaar@comunidad.unam.mx (A.A.-R.); 2 Instituto de Biología, Universidad Nacional Autónoma de México, Ciudad de México 04510, Mexico; pvelez@ib.unam.mx; 3 Departamento de Microbiología, Centro de Investigación Científica y de Educación Superior de Ensenada (CICESE), Ensenada, Baja California 22860, Mexico; riquelme@cicese.mx; 4 Institute for Tuberculosis Research, College of Pharmacy, University of Illinois at Chicago, Chicago, IL 60612, USA; baojie@uic.edu (B.W.); rma20@uic.edu (R.M.); mallique@uic.edu (M.Q.); sgf@uic.edu (S.G.F.)

**Keywords:** marine fungi, deep-sea sediments, chemical diversity, metabolomics, antimicrobial activity

## Abstract

A collection of 29 cultivable fungal strains isolated from deep-sea sediments of the Gulf of Mexico were cultivated under the “one strain, many compounds” approach to explore their chemical diversity and antimicrobial potential. From the 87 extracts tested, over 50% showed antimicrobial activity, and the most active ones were those from cultures grown at 4 °C in darkness for 60 days (resembling deep-sea temperature). PCA analysis of the LC-MS data of all the extracts confirmed that culture temperature is the primary factor in the variation of the 4462 metabolite features, accounting for 21.3% of the variation. The bioactivity-guided and conventional chemical studies of selected fungal strains allowed the identification of several active and specialized metabolites. Finally, metabolomics analysis by GNPS molecular networking and manual dereplication revealed the biosynthetic potential of these species to produce interesting chemistry. This work uncovers the chemical and biological study of marine-derived fungal strains from deep-sea sediments of the Gulf of Mexico.

## 1. Introduction

Microorganisms are the most abundant and diverse living organisms on the planet, contributing to around 60% of the total of Earth’s biomass [1]. Marine microbial diversity plays an important role in the global cycling of nutrients, matter, and energy [2]. Although the number of marine microorganisms is still unknown, especially due to the complications of laboratory cultivation, new methodologies (multi-omics) have emerged to address this question and others related to the different types of organisms, their functional roles, global distribution, and adaptation to varying environmental conditions [2].

Microbes are true master chemists, capable of carrying out the most diverse and complex chemical reactions, and microbial natural products continue to be an important source of new drugs and structural prototypes for the development of new therapeutic agents [2,3].

Endemic fungi and other ubiquitous deep-sea species are adapted to constant darkness, high hydrostatic pressures, microaerophilic conditions, low temperatures (2–4 °C, except for vent systems), low pH, limited nutrients, and the combination of these parameters [4,5,6]. In addition, it is probable that these microorganisms produce structural molecules such as lipids, enzymes, and biopolymers with unique properties that allow them to thrive under extreme conditions [6]. There are about 10,000 species of marine fungi belonging mainly to the *Ascomycota* and *Basidiomycota*, estimated in sediments, water columns, driftwood, sessile and mobile invertebrates, algae, and marine mammals [7]. This number is underestimated, as many locations and habitats remain unexplored.

The Gulf of Mexico (GoM) is a large reservoir of scantly studied marine microorganisms [8]. In the last decade, culture-dependent and -independent analyses have been carried out to explore the microbial diversity of the waters and sediments of the GoM. Moreover, the role of these microorganisms in the bioremediation of the ocean is of great importance, e.g., in water and sediments contaminated with mono and polycyclic aromatic hydrocarbons, intermediate chain alkanes, methane, and other gases released during accidental oil spills, mainly by synthesizing enzymes that transforms these products into harmless and less persistent molecules [8,9,10,11,12,13,14,15]. Marine fungi have also shown the ability to produce secondary metabolites that allow them to survive against predators [5]. Finally, the potential ecological participation of these microorganisms as pathogens of animals with commercial importance has been revised [16].

Metagenomic analysis has shown that the GoM contains an unusual fungal diversity compared to marine sediments from other regions in the world. Interestingly, metabolically active fungi have not been studied as deeply as marine bacteria [8,9,17,18]. Recently, a series of cultivable fungi from sediments of two oil-drilling deep-sea oil reserves in the GoM were studied for their capacity to grow in media with hexadecane and 1-hexadecene as the only source of carbon [17]. These organisms showed different gene expression profiles. Key hydrocarbonoclastic taxa displayed up-regulated genes involved in transmembrane transport, metabolism of carbohydrates, and nitric oxide pathways. Based on these findings, the GoM represents an important source of fungi with potential for the discovery of active specialized metabolites [17,19].

With this study, we investigated the chemical diversity and antimicrobial potential of 29 cultivable fungi isolated from deep-sea sediments of the GoM. Their extracts obtained from cultures prepared under the “one strain, many compounds” (OSMAC) approach were screened against a set of clinically relevant pathogens. Two active and two inactive species were selected for bioactivity-guided and conventional chemical analysis, respectively. This resulted in the isolation of two benzochromenones, four benzodiazepines, one cytochalasin, and one benzylisoquinoline alkaloid. Additionally, metabolomic analysis by Global Natural Products Social (GNPS) molecular networking and manual dereplication, using the high-resolution mass spectrometry (HRMS-MS/MS) data of their extracts, resulted in the annotation of 15 secondary metabolites. Finally, some of the isolated compounds showed activity against ESKAPE pathogens and tuberculous and non-tuberculous *Mycobacteria*.

## 2. Results and Discussion

**Figure 1:**
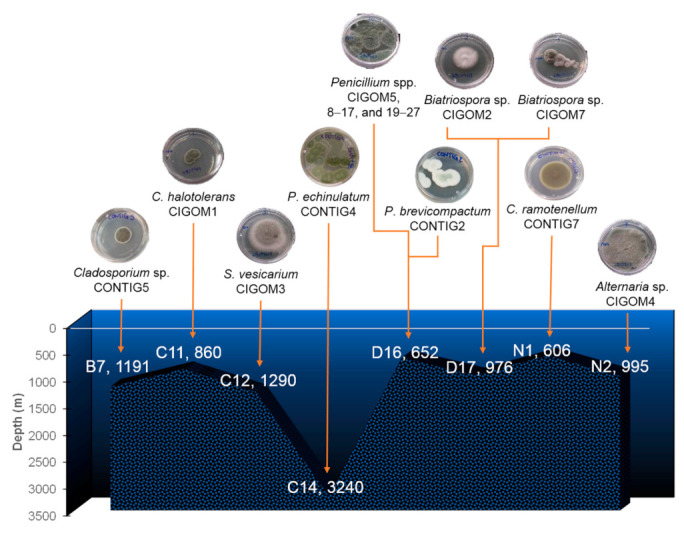
Marine fungi from deep-sea sediments of the GoM.

As a part of a program to explore the chemical diversity and antimicrobial potential of fungal species isolated from unexplored areas of Mexico, a series of 29 cultivable fungal strains from marine sediments of the GoM [17] were grown under laboratory conditions. These organisms belong to the genera *Penicillium*, *Cladosporium*, *Stemphylium*, *Biatriospora*, and *Alternaria* (Figure 1 and Table 1). Fungi of the *Penicillium* genus were the most abundant in the samples: *P. echinulatum* CONTIG4 was obtained from the C14 (Coatzacoalcos) site at a depth of 3240 m, the deepest sampled station. *P. brevicompactum* CONTIG2 and *Penicillium* spp. CIGOM5, CIGOM8-CIGOM17, and CIGOM19-CIGOM27 were obtained from the D16 (Coatzacoalcos) site at a depth of 652 m. In addition, *Cladosporium* sp. CONTIG5, *C. halotolerans* CIGOM1, and *C. ramotenellum* CONTIG7 were obtained from the B7 (Perdido), C11 (Coatzacoalcos), and N1 (Perdido) stations at 1191, 860, and 606 m, respectively. Finally, *Biatriospora* spp. CIGOM2 and CIGOM7 were obtained from the D17 (Coatzacoalcos) station at 976 m, and *Alternaria* sp. CIGOM4 from N2 (Perdido) at 995 m. All strains were maintained in potato dextrose agar (PDA) medium at room temperature (RT, between 18–22 °C) until being used for small- and large-scale cultures.

### 2.1. Antimicrobial Screening of the Fungal Collection and Effects of Culture Conditions

In the “one strain, many compounds” (OSMAC) approach, the growth conditions of fungal strains (medium composition, pH, O_2_, temperature, etc.) are modified to activate cryptic or silent biosynthetic pathways [20]. Different temperatures (RT, 20 °C, and 4 °C) and light/darkness ratios were used for the growth of the 29 strains in rice medium (for details, see Section 3). The defatted CHCl_3_-MeOH (1:1) extracts of the small-scale cultures were tested against the bacteria *Escherichia coli* ATCC 10536, *Salmonella typhi* ATCC 9992V, *Pseudomonas aeruginosa* ATCC 27853, *Staphylococcus aureus* ATCC 25923 methicillin-susceptible (MSSA), and *Bacillus subtilis* ATCC 6633, and the yeast *Candida albicans* ATCC 10231, at 200 µg/mL and 20 µg/mL. From the 87 extracts evaluated, 54 (62%) showed antimicrobial activity (Table 2 and Table S1). The most active ones (12 extracts) were from cultures grown at 20 °C in darkness for 30 d or 4 °C in darkness for 60 d. Extracts from *Alternaria* sp. CIGOM4, *C. ramotenellum* CONTIG7, and *P. echinulatum* CONTIG4 showed the highest antimicrobial potential. *Alternaria* sp. CIGOM4, cultivated at 4 °C in darkness for 60 d, completely inhibited *B. subtilis* at 20 µg/mL, the lowest evaluated concentration, and MSSA at 200 µg/mL, while the extract obtained from the 20 °C darkness 30 d culture, was active against both bacteria at 200 µg/mL. Similarly, the extract of *P. echinulatum* CONTIG4 grown at 20 °C in darkness for 30 d showed antimicrobial activity against *E. coli*, *S. typhi*, and MSSA at 200 µg/mL. Finally, *C. ramotenellum* CONTIG7 showed antimicrobial activity on Gram-positive bacteria when grown at 20 °C in darkness for 30 d and at RT with light/darkness 12/12 h for 21 d (Table 2).

**Figure 2:**
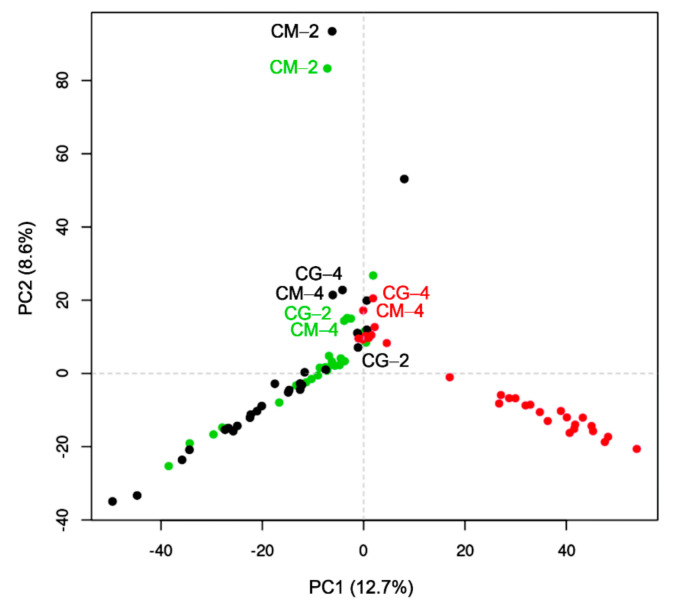
Effect of incubation temperature on the overall metabolites profile determined by PCA analysis of all GoM fungal samples (strains CIGOM (CM) and CONTIG (CG); colors represent growth temperature: red, 4 °C; black, 20 °C; green, RT).

Interestingly, fungal extracts of a single strain prepared under different growth conditions showed different antimicrobial activity. In marine environments, temperature and light decrease with depth. As observed in Table 2, the antimicrobial activity of the evaluated fungal extracts increased when growth parameters resembled the undersea conditions. To explore the relative impact of the culture conditions in metabolite production, the 87 extracts were subjected to ultra-performance liquid chromatography coupled to photodiode array detection and electrospray ionization tandem high resolution mass spectrometry (UPLC-PDA-HRESIMS-MS/MS) analysis followed by principal component analysis (PCA), using temperature as the determinant variable (Figure 2).

PCA was performed with 4462 metabolite features retained after blank removal (Table S2). Data showed statistically significant clustering depending on the culture temperature, which is a primary factor in the metabolite’s profiles and accounts for 21.3% of the overall chemical variation (Figure 2). Cultures grown at 4 °C had very slow growth rates, and as expected, metabolites with distinctive features were grouped together (red points) and separated from those obtained from the same strains grown at 20 °C (black points) and RT (green points; Figure 2). For *Biatriospora* sp. CIGOM2 (CM−2) cultivated at 20 °C and RT, there is a grouping separated from the rest of the evaluated species, indicating notable differences in its chemical profile (Figure 2).

For the bioactivity-guided chemical study, scaled-up cultures of two of the most active fungi, *Alternaria* sp. CIGOM4 and *P. echinulatum* CONTIG4, were grown at 4 °C and 20 °C in darkness for 60 and 30 d, respectively. These strains were also cultivated in rice medium supplemented with a Czapek-Dox solution or artificial marine water to explore their metabolic profile under salty conditions. Salinity is a known abiotic factor that can trigger secondary metabolite production [5]. In addition, to further explore the chemical diversity of the fungal collection in addition to the biological potential, the species *Biatriospora* sp. CIGOM2 and *Penicillium* sp. CIGOM10 were subjected to chemical analysis. For all selected species, changes in their main secondary metabolites were established by UPLC-PDA-HRESIMS-MS/MS analysis, and their chemical diversity was explored using the Global Natural Products Social (GNPS) molecular networking platform and manual dereplication analysis [21].

### 2.2. Chemical Study of Selected Fungal Strains

**Figure 3:**
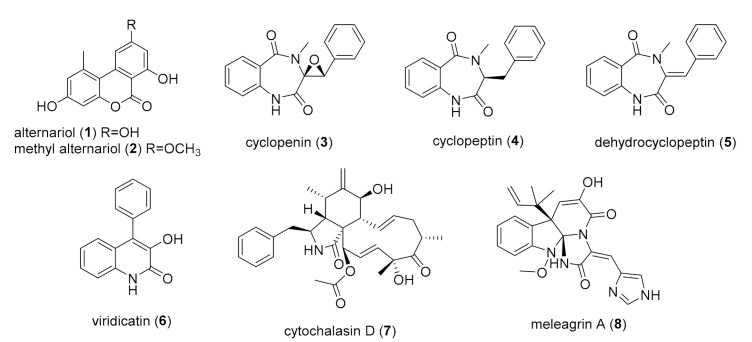
Compounds isolated from strains *Alternaria* sp. CIGOM4, *P. echinulatum* CONTIG4, *Biatriospora* sp. CIGOM2, and *Penicllium* sp. CIGOM10.

The bioactivity-guided chemical analysis of the scaled-up extracts of *Alternaria* sp. CIGOM4 and *P. echinulatum* CONTIG4, and the conventional chemical study of *Biatriospora* sp. CIGOM2, and *Penicllium* sp. CIGOM10, yielded eight compounds (Figure 3). Briefly, the extracts were fractionated by flash chromatography using normal-phase columns and a mobile phase composed of *n*-hexane, CHCl_3_, EtOAc, and CH_3_OH mixtures. The antimicrobial activity of the fractions was assessed against the same panel of microorganisms (Table S3). Active and some non-active fractions were separated by reverse-phase HPLC (preparative and semipreparative level) on CH_3_CN-0.1% aqueous formic acid gradient mobile phase (for details, see Section 3). All compounds were characterized by comparison of their spectroscopic (NMR) and spectrometric (HRESIMS-MS/MS) data with those reported in literature (Table S4 and Figures S1–S11, see Supplementary Materials) [22,23,24,25,26,27,28].

Benzochromenone alternariol (**1**) and its methyl-derivative (**2**) were obtained from *Alternaria* sp. CIGOM4 antimicrobial fractions, F7 and F4, respectively. The benzodiazepines cyclopenin (**3**), cyclopeptin (**4**), and 9,10-dehydrocyclopeptin (**5**), and the hydroxyquinolone, viridicatin (**6**) were isolated from the antimicrobial fraction F5 and the inactive fraction F6 of *P. echinulatum* CONTIG4. Finally, the mycotoxin cytochalasin D (**7**) and the benzylisoquinoline alkaloid, meleagrin A (**8**) were isolated from the inactive extracts of *Biatriospora* sp. CIGOM2 and *Penicillium* sp. CIOGM10, respectively (Figure 3; for details, see Section 3).

### 2.3. Metabolomic Study of Selected GoM Fungal Strains

**Figure 4:**
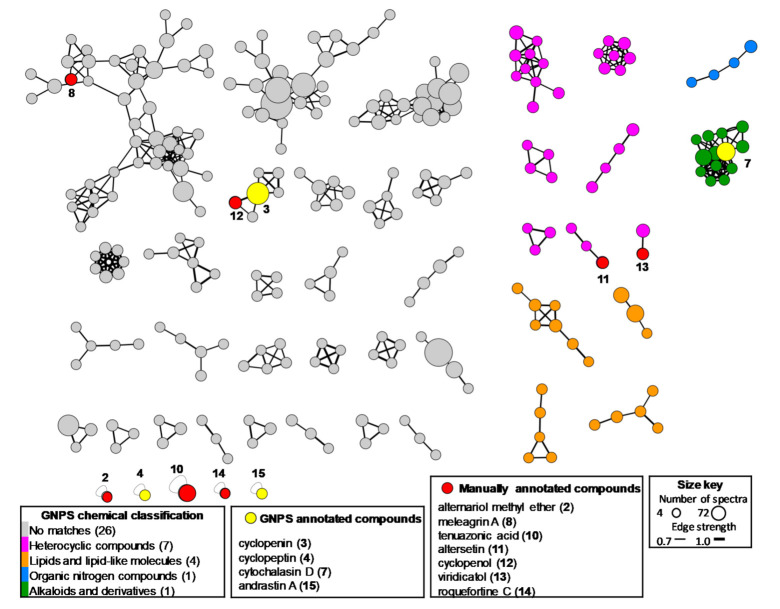
Feature-based GNPS analysis of the metabolites produced by *Alternaria* sp. CIGOM4, *P. echinulatum* CONTIG4, *Biatriospora* sp. CIGOM2 and *Penicillium* sp. CIGOM10. Edge strength indicates the chemical similarity between MS/MS spectra. Compounds annotated manually and by GNPS are indicated in boxes with arrows pointing to the corresponding node (mass accuracy < 5 ppm).

To explore the metabolic diversity of the selected fungi *Alternaria* sp. CIGOM4, *P. echinulatum* CONTIG4, *Biatriospora* sp. CIGOM2, and *Penicllium* sp. CIGOM10, cultivated under different conditions, UPLC-PDA-HRESIMS-MS/MS data of their extracts were subjected to GNPS molecular networking and manual dereplication analysis. First, the molecular network of all four strains displayed 38 clusters containing at least three nodes grouped in four subclasses (Figure 4). From this, 26 non-matching clusters were observed, which probably correlates to the unique chemistry of these strains. Clusters 7 and 4 were annotated as heterocyclic and lipids/lipid-like molecules, respectively; one in the alkaloids and derivatives category; and one in the organic nitrogen compounds category (Figure 4). GNPS automatically annotated compounds (Table 3) were cyclopenin (**3**) and cyclopeptin (**4**), isolated from *P. echinulatum* CONTIG4; cytochalasin D (**7**), isolated from *Biatriospora* sp. CIGOM2; and andrastin A (**15**), a farnesyltransferase inhibitor isolated from several *Penicillium* species. Furthermore, methyl alternariol (**2**), meleagrin A (**8**), tenuazonic acid (**10**), altersetin (**11**), cyclopenol (**12**), viridicatol (**13**), and roquefortine C (**14**) were manually dereplicated and annotated by comparison of their UV-absorption maxima and HRMS-MS/MS data against isolated or previously reported compounds, at confidence levels 1 and 2 according to the metabolomics standards initiative and exact mass accuracy < 5 ppm [20,29,30].

Next, changes in the production of secondary metabolites for the selected fungal strains under the OSMAC cultivation-based approach [31], were assessed by comparison with the UPLC-PDA-HRESIMS-MS/MS profiles and by GNPS molecular networking analysis of the extracts obtained from each growth condition.

**Figure 5:**
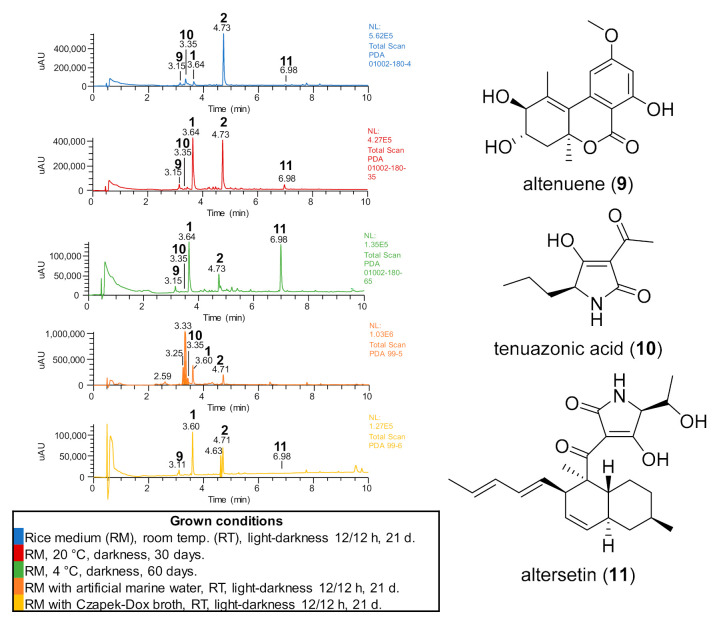
UPLC-PDA profiles comparison of *Alternaria* sp. CIGOM4 extracts obtained under different growth conditions (**left**). Compounds identified by manual dereplication (**right**).

In the case of *Alternaria* sp. CIGOM4, the main metabolites alternariol (**1**) and its methyl ether derivative (**2**) were observed in all growth conditions (Figure 5). The culture grown at 20 °C contained the highest concentration of these compounds at a 1:1 ratio. Interestingly, at RT, the fungus produced compound **2** almost exclusively, while at 4 °C, altersetin (**11**) and **1** were the main products. In addition, minor metabolites altenuene (**9**) and tenuazonic acid (**10**) were produced in rice medium, but they were not observed when the fungus was grown with artificial sea water or Czapek-Dox media (Figure 5).

**Figure 6:**
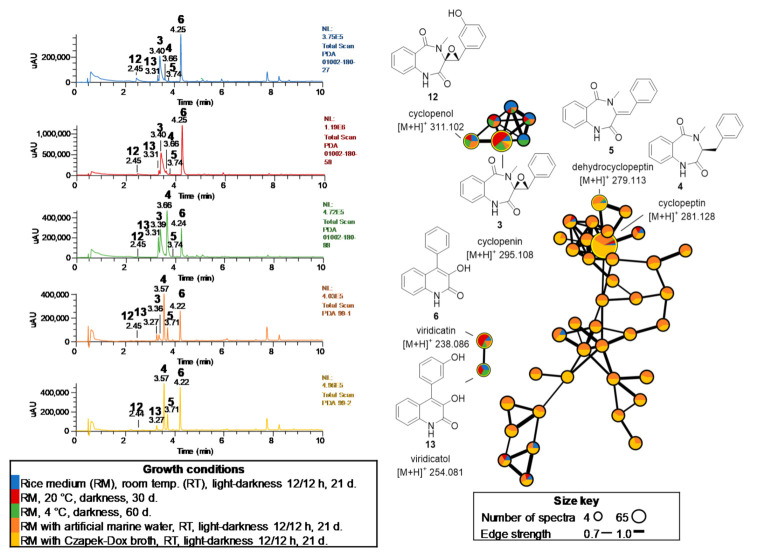
UPLC-PDA profiles comparison and GNPS molecular networking of *P. echinulatum* CONTIG4 extracts obtained under different growth conditions.

The strain *P. echinulatum* CONTIG4 showed notable biosynthetic potential as it produced benzodiazepines **3**–**5** and the quinolone **6**. In addition to these compounds, manual dereplication of the extracts allowed to identify cyclopenol (**12**) and viridicatol (**13**), phenolic derivatives of **3** and **6**, respectively (Figure 6). Comparison of the UPLC-PDA-HRESIMS-MS/MS profiles of the extracts revealed that viridicatin (**6**) was produced at high levels in all conditions, and 9,10-dehydrocyclopeptin (**5**) was overproduced when the strain was grown at 4 °C or in salty conditions. Compound **5** was also produced in salty medium, while **3** decreases in these extracts to undetectable levels (Figure 6). GNPS analysis of these extracts grouped all these biological active alkaloids into three clusters, where nodes connected to each other correspond to structurally related compounds (Figure 6).

**Figure 7:**
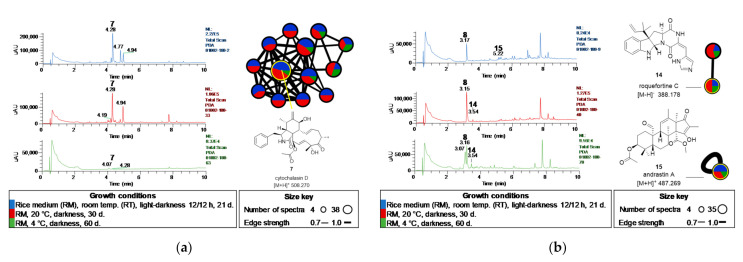
UPLC-PDA profiles comparison and GNPS molecular networkings of (**a**) *Biatriospora* sp. CIGOM 2 (**b**) and *Penicillium* sp. CIGOM10 extracts obtained under different growth conditions.

Finally, a comparison of the extracts of *Biatriospora* sp. CIGOM 2 and *Penicillium* sp. CIGOM10 obtained under different growth conditions is shown in Figure 7. The strain CIGOM2 yielded the mycotoxin cytochalasin D (**7**) as its main product; however, when the fungus was grown at 4 °C, this compound was barely produced (Figure 7a). Additionally, peaks observed in the RT and 20 °C extracts at a retention time (t_R_) of 4.77 min (this peak disappeared fungus was grown at 20 °C) and 4.94 min had the same molecular ion as **7**. In the Dictionary of Natural Products, there are nine isomeric cytochalasins derivatives with this weight: cytochalasin C, M and Q, 19,20-epoxy-18-deoxycytochalasin C, 19,20-epoxy-18-deoxycytochalasin Q, xylobovatin, chaetoconvosin B, and phomopsichalasin D, that could be correlate to compounds at t_R_ 4.77 min and 4.94 min. In addition, the GNPS cluster of the annotation of **7**, showed all MS/MS fragments reported on the MassBank (record FIO00864; https://massbank.eu/MassBank/, accessed on 26 November 2021) for this product (Figure 7a). In the case of Penicillium sp. CIGOM10 (Figure 7b), meleagrin A (**8**) was the major metabolite in all conditions. By manual dereplication and GNPS annotation, andrastin A (**15**) was detected only in the RT extract, while roquefortine C (**14**), a meleagrin A (**8**) precursor, was detected when the strain was grown at lower temperatures (Figure 7b).

### 2.4. Biological Activity of ***1***–***8***

The in vitro antibacterial activity of isolated compounds **1**–**8** was assessed against a panel of ESKAPE pathogens using the microdilution assay (Table S5) [32], and tuberculous and non-tuberculous *Mycobacteria* using the microplate Alamar blue (MABA) and low oxygen recovery (LORA) assays (Table S6) [33,34]. Additionally, their cytotoxicity was established against Vero cell lines (Table S6) [35]. From this, only compounds **1**, **6**, and **8** showed biological activity (Table 4).

Alternariol (**1**) showed total inhibition of *S. aureus* methicillin-resistant (MRSA) strain when tested at 387.3 μM, and it was 10 times more potent (38.7 μM) when tested against a methicillin-susceptible strain (Table 4). This compound also showed 87% growth inhibition of *M. tuberculosis* at 50 μg/mL. In previous reports, **1** showed important activity against a non-resistant *Bacillus subtilis* ATCC 6633 with minimum inhibition concentration (MIC) of 33.3 μM [36]. Thus, the activity observed in *Alternaria* sp. CIGOM4 extracts of cultures incubated at 4 °C and 20 °C (Table 2) is most likely due to the alternariol (**1**).

Viridicatin (**6**) did not show antimicrobial activity against *S. aureus* strains but inhibited the growth of *M. tuberculosis* with MIC of 43.8 μM (Table 2) [37,38]. This compound was inactive against Vero cell lines at the tested concentration (Table 4).

Finally, meleagrin A (**8**), isolated from CIGOM 10, showed partial inhibition against MSSA at the highest concentration tested (Table 4). In previous studies, this compound demonstrated antibiofilm activity vs. *S. aureus* ATCC 29213, with 87.1% of inhibition at 69.2 µM [39]. It also inhibited FabI, an isoform of enoyl-ACP reductase that participates in the fatty acid biosynthesis in multidrug-resistant bacteria [40], and antimicrobial activity against different microorganisms in the paper-disk diffusion assay [39]. Lastly, this compound was weakly active against *Micrococcus luteus* DSMZ 1605 [39]. In our assays, meleagrin A (**8**) displayed anti-*M. tuberculosis* and *M avium* activity with MIC of 48.0 and 12.3 μM, respectively, and no cytotoxic activity against Vero cell line was observed (Table 4).

## 3. Materials and Methods

### 3.1. Strains, Cultures, and Extract Preparations

Twenty-nine fungal strains were isolated from deep-sea sediment samples collected from eight stations of the GoM during the Metagenomica-Malla Fina cruise (MET-I) and Metagenomics (MET-II) campaigns in 2016 and 2017, respectively, onboard the research vessel Justo Sierra of UNAM (Table 1) [17,18]. Each axenic culture in the PDA plates was transferred to (1% of yeast extract, 2% of soy peptone, 2% of dextrose) medium and incubated for 5 d at RT in a shaker at 120 rpm. All inoculums were transferred to 250 mL Erlenmeyer flasks with rice medium (15 g/30 mL of deionized water) and maintained under three different conditions: (1) RT with light-darkness 12/12 h for 21 d; (2) 20 °C in darkness for 30 d; and (3) 4° C in darkness for 60 d. After growth, each fungus was extracted with 60 mL of 1:1 CH_3_OH-CHCl_3_, shaken on an orbital shaker at 100 rpm, and filtered. Then, 60 mL of CHCl_3_ and 120 mL of H_2_O were added to the filtrates and mixed again. The organic layers were separated in a separatory funnel and dried under reduced pressure. The residues were dissolved in 60 mL of 1:1 CH_3_CN-CH_3_OH and defatted with the same volume of *n*-hexane. Defatted extracts were preserved at room temperature until use [41,42]. Scale-up cultures and extracts of selected strains were prepared using the same methodology as the small-scale cultures but in 150 g of rice (300 mL of deionized water in a 2.8 L Fernbach flask) and solvent volumes adjusted accordingly. Finally, selected active strains were also grown in rice with Czapek-Dox solution (100 g of rice with 200 mL of Czapek-Dox solution composed of sucrose, 30 g/L; NaNO_3_, 2 g/L; K_2_HPO_4_, 1 g/L; MgSO_4_, 0.5 g/L, KCl, 0.5 g/L; and FeSO_4_, 0.01 g/L; pH 7.3 at 25 °C) or artificial marine water (32 g/L of Instant Ocean Sea Salt) instead of deionized water, in a 2.8 L Fernbach flask at RT with light-darkness 12/12 h for 21 d [42].

### 3.2. In Vitro Biological Testing

Defatted extracts, fractions, and pure compounds were tested for antibacterial activity using the broth dilution methods with MTT, following the standard and approved procedures published by the Clinical and Laboratory Standards Institute (CLSI) for microbial testing [32]. DMSO (2.0%) was used to dissolve all samples. Target bacteria used in the assays (Table 4, Table S1, Table S3 and Table S5) were *Bacillus subtilis* ATCC 6633, *Staphylococcus aureus* methicillin-resistant ATCC 43300 (MRSA) and methicillin-susceptible ATCC 25923 (MSSA), *Escherichia coli* ATCC 10536, *Salmonella typhi* ATCC 9992V, *Pseudomonas aeruginosa* ATCC 27853, *Enterococcus faecalis* vancomycin-resistant ATCC 51299 (VREF) and vancomycin-susceptible ATCC 29212 (VSEF), *Klebsiella aerogenes* ATCC 13048, *Enterobacter cloacae* ATCC 700324, *Klebsiella pneumoniae* ATCC 700603, and *Acinetobacter baumannii* antibiotic resistant (clinical isolated strain 564) and susceptible ATCC17978, and the yeast *Candida albicans* ATCC 10231. The bioassays were carried out in 96-well plates in triplicate at concentrations of 200 μg/mL and 20 μg/mL for extracts and fractions, and 100 μg/mL and 10 μg/mL of pure compounds. Pure compounds **1**–**8** were also tested against *M. tuberculosis* H37Rv strain under both aerobic (replicating) and anaerobic (nonreplicating) conditions using MABA and LORA (Table S6) [33]. Compounds **6** and **8**, with activity above 90% of inhibition of the growth of *M. tuberculosis* in the MABA, were further tested against *M. abscessus* ATCC 19977, *M. chelonae* ATCC 35752, *M. marinum* ATCC 927, *M. avium* ATCC 15769, and *M. kansasii* ATCC 12478, using MABA [33,34] and the Vero cell lines ATCC CRL-81 using (3-(4,5-dimethylthiazol-2-yl)-2,5-diphenyltetrazolium bromide (MTT) assay (Table S6) [35]. For the latter assay, 0.6 mM of resazurin was used and the absorbance was recorded at 530 nm (excitation) and 590 nm (emission). Positive controls for all assays are indicated in each table of results.

### 3.3. Chemical Study of Selected Fungal Strains

The extract (194.0 mg) of *Alternaria* sp. CIGOM4 was fractionated via flash chromatography on a RediSep RF Gold Si-gel column (4.0 g of Si-gel; Teledyne Inc., Thousand Oaks, CA, USA) using sequential mixtures of *n*-hexane-CHCl3-AcOEt-MeOH. Nine fractions were obtained according to their UV and ELSD profiles. Alternariol (**1**; 35.5 mg) was obtained pure from fraction 7. Fraction 4 was subjected to preparative HPLC (Gemini C18 column 250 mm × 21.2 mm I.D., 5.0 μm, 100 Å; Phenomenex Inc., Torrance, CA, USA) with a gradient system from 30:70 CH_3_CN–0.1% aqueous formic acid to 100 of CH_3_CN in 15 min at 21.24 mL/min, yielding 2.8 mg of alternariol 5-*O*-methyl ether (**2**; *t*_R_ = 11.8 min).

From *P. echinulatum* CONTIG4, extract (2.1 g) was fractionated via flash chromatography on a RediSep RF Gold Si-gel column (40 g of Si-gel) using sequential mixtures of *n*-hexane-CHCl3-AcOEt-MeOH. Thirteen primary fractions were obtained according to their UV and ELSD profiles. Fractions 5 (174.8 mg), 6 (109.0 mg), and 7 (43.4 mg) were subjected to preparative HPLC (Gemini C18 column 250 mm × 21.2 mm I.D., 5.0 μm, 100 Å) with a gradient system from 30:70 CH_3_CN–0.1% aqueous formic acid to 100 of CH_3_CN in 15 min at 21.24 mL/min, yielding cyclopenin (**3**; 5.5 mg, *t*_R_ = 9.197 min), cyclopeptin (**4**; 46.6 mg, *t*_R_ = 9.754 min), dehydrocyclopetin (**5**; 6.2 mg, *t*_R_ = 10.236 min), and viridicatin (**6**; 14.1 mg, *t*_R_ = 12.044 min).

The extract (38.4 g) of *Biatriospora* sp. CIGOM 2 was fractioned via flash chromatography on a RediSep RF Gold Si-gel column (4 g of Si-gel) using sequential mixtures of *n*-hexane-CHCl_3_-AcOEt-MeOH. Five primary fractions were obtained according to their UV and ELSD profiles. Fraction four was subject to semipreparative HPLC (Gemini C18 column 250 mm × 10.0 mm I.D., 5.0 μm, 100 Å) with a gradient system from 15:85 CH_3_CN–0.1% aqueous formic acid to 100 of CH_3_CN in 15 min at 4.72 mL/min, yielding cytochalasin D (**7**; 1.7 mg, *t*_R_ = 13.95 min).

Finally, the extract (1.0 g) of *Penicillium* sp. CIGOM 10 was fractionated via flash chromatography on RediSep RF Gold Si-gel column (40 g of Si-gel) using sequential mixtures of *n*-hexane-CHCl_3_-AcOEt-MeOH. Twelve fractions were obtained according to their UV and ELSD profiles. Fraction 10 (83.6 mg) was subject to preparative HPLC (Gemini C18 column 250 mm × 21.2 mm I.D., 5.0 μm, 100 Å) with a gradient system from 15:85 CH_3_CN–0.1% aqueous formic acid to 100 of CH_3_CN in 15 min at 21.24 mL/min, yielding meleagrin A (**8**; 14.4 mg, *t*_R_ = 6.30 min).

### 3.4. LC-MS/MS, Untargeted Metabolomic and Molecular Network Analysis

Extracts (1 mg/mL), fractions (1 mg/mL), and pure compounds (0.1 mg/mL) were analyzed on an Acquity UPLC (Waters Corp., Milford, MA, USA) coupled to a Q Exactive Plus (Thermo Fisher Scientific, Waltham, MA, USA) mass spectrometer. LC analysis was performed on an Acquity BEH C18 column (Waters 50 mm × 2.1 mm I.D., 1.7 μm, 130 Å) at 40 °C, with a gradient system from 15:85 CH_3_CN–0.1% aqueous formic acid to 100% of CH_3_CN in 8 min, then held for 1.5 min with CH_3_CN and returned to the starting conditions, flow rate of 0.3 mL/min, and injection volume of 3.0 μL. HRMS-MS/MS data were obtained using an ESI source (positive and negative modes) at a full scan range (*m*/*z* 150–2000), with the following settings: capillary voltage, 5 V; capillary temperature, 300 C; tube lens offset, 35 V; spray voltage, 3.80 kV; sheath and auxiliary gas flow, 30 arbitrary units. [30]. Then, MS raw data of all samples were converted to mzXML format using MS Converter of ProteoWizard tool. PCA analysis was performed from the MS data (molecular features after blank removal) using R software (version 4.0.5) with the package FactoMineR [43]. Metabolomic analysis by GNPS molecular networking of all extracts and for the selected fungal strains was assessed using the standard protocol [21] with the following parameters: precursor ion mass tolerance, 0.01 Da; fragment ion mass tolerance, 0.02 Da; minimum cosine score and score threshold, 0.7; minimum matched fragment ions, cluster size, and library search minimum matched peaks, 4.0; and maximum connected component size and maximum analog search mass difference, 100. MolNetEnhancer tool was applied for chemical classification [44]. Molecular networks were visualized with Cytoscape 3.8.1 [45]. Finally, manual dereplication was assessed using UV-absorption maxima and HRMS-MS/MS data against MS/MS data of **1**–**8** and by comparison with those reported in the Dictionary of Natural products [46], SciFinder [47], and an *in-house* mycotoxins database. The annotation of isolated compounds **1**–**8** and annotated **9–15** was at confidence level 1 and 2, respectively, according to the metabolomics standards initiative [29] and exact mass accuracy <5 ppm.

### 3.5. Data Availability

LC-MS/MS data can be accessed at MassIVE (accession no. MSV000088218; accessed on 26 November 2021). The molecular network of selected fungi can be accessed at http://gnps.ucsd.edu/ProteoSAFe/status.jsp?task=1e7a7e47a5c54413a52ef96708565aaf (MolNetEnhancer analysis; accessed on 26 November 2021), molecular network of CONTIG4 at http://gnps.ucsd.edu/ProteoSAFe/status.jsp?task=3b276ea9c0354dd4975472b560e2a6f7 (accessed on 26 November 2021), molecular network of CIGOM2 at http://gnps.ucsd.edu/ProteoSAFe/status.jsp?task=fd2034712bbd4bb3b72c3edea4e725ef (accessed on 26 November 2021), molecular network of CIGOM10 at http://gnps.ucsd.edu/ProteoSAFe/status.jsp?task=a9a38aed67dc4e69a8caf34d5e1cb2fb (accessed on 26 November 2021).

## 4. Conclusions

This work advances our chemical and biological knowledge of a series cultivable fungal strains isolated from deep-sea sediments of the Gulf of Mexico, an important and poorly studied ecosystem with significant environmental damage as a result of anthropogenic activities. Under the OSMAC approach, the chemical and antimicrobial potential of these strains was exposed. Interestingly, over 50% of the extracts tested showed antimicrobial activity. The most active were the ones grown under conditions that resemble the deep-sea environment. PCA analysis confirmed that culture temperature is the main factor of chemical variation. The chemical study of selected fungal strains, together with GNPS molecular networking and untargeted metabolomics, allowed the biosynthetic potential of these species to produce interesting chemistry to be discovered. Although several strains did not show biological activity, their potential to produce new chemistry remains to be investigated.

## Figures and Tables

**Table 1:** Sampling stations and depths of sediments collection in the GoM.

Station	Depth (m)	Strain
B7 (Perdido)	1191	*Cladosporium* sp. CONTIG5
C11 (Coatzacoalcos)	860	*Cladosporium halotolerans* CIGOM1
C12 (Coatzacoalcos)	1290	*Stemphylium vesicarium* CIGOM3
C14 (Coatzacoalcos)	3240	*Penicillium echinulatum* CONTIG4
D16 (Coatzacoalcos)	652	*Penicillium* spp. CIGOM5, 8–17, and 19–27 *P. brevicompactum* CONTIG2
D17 (Coatzacoalcos)	976	*Biatriospora* sp. CIGOM2 y CIGOM7
N1 (Perdido)	606	*Cladosporium ramotenellum* CONTIG7
N2 (Perdido)	995	*Alternaria* sp. CIGOM4

**Table 2:** Active antimicrobial extracts of marine fungi from the GoM.

Strain	Extract Condition	*E. coli* ATCC 10536	*S. typhi* ATCC 9992v	MSSA	*B. subtilis* ATCC 6633
*P.echinulatum* CONTIG4	B				
*C. ramotenellum* CONTIG7	A B				
*S. vesicarium* CIGOM3	C				
*Alternaria* sp. CIGOM4	B				
	C				
*Biatriospora* sp. CIGOM7	C				
*Penicillium* sp. CIGOM16	C				
*Penicillium* sp. CIGOM17	C				
*Penicillium* sp. CIGOM20	C				
*Penicillium* sp. CIGOM21	C				
*Penicillium* sp. CIGOM22	C				
*Penicillium* sp. CIGOM24	A				
*Penicillium* sp. CIGOM26	A				
	C				
MIC ampicillin (μg/mL)		1.6	0.4	0.1	16.3
**Inhibition level**					
	Total inhibition at 200 μg/mL and 20 μg/mL.				
	Total inhibition at 200 μg/mL.				
	Partial inhibition at 200 μg/mL and 20 μg/mL.				
	Partial inhibition at 200 μg/mL.				
	No inhibition.				

Rice medium at (A) RT with light/darkness 12/12 h for 21 d; (B) 20 °C in darkness for 30 d; and (C) 4 °C in darkness for 60 d.

**Table 3:** Chemical annotation by GNPS and by comparison with isolated compounds from selected GoM fungal strains in the molecular networking.

Compound	Adduct	Observed Ion ^a^	Molecular Formula	Exact Mass ^d^	Mass Accuracy (ppm)
Alternariol (**1**)	[M − H]^−^	257.045	C_14_H_10_O_5_	257.0452	−1.3
Alternariol methyl ether (**2**)	[M + H]^+^	273.076	C_15_H_12_O_5_	273.0755	−0.9
Cyclopenin (**3**)	[M + H]^+^	295.108	C_17_H_14_N_2_O_3_	295.1074	−1.10
Cyclopeptin (**4**)	[M + H]^+^	281.128	C_17_H_16_N_2_O_2_	281.1282	−0.9
Dehydrocyclopeptin (**5**)	[M + H]^+^	279.113	C_17_H_14_N_2_O_2_	279.1126	−0.7
Viridicatin (**6**)	[M + H]^+^	238.086	C_15_H_11_NO_2_	238.0860	−1.1
Cytochalasin D (**7**)	[M + H]^+^	508.270	C_30_H_37_NO_6_	508.2689	−0.7
Meleagrin A (**8**)	[M + H]^+^	434.182	C_23_H_23_N_5_O_4_	434.1820	−0.6
Altenuene (**9**) ^b^	[M + H]^+^	293.102	C_15_H_16_O_6_	293.1017	−0.9
Tenuazonic acid (**10**) ^b^	[M + H]^+^	198.113	C_10_H_15_NO_3_	198.1123	−0.9
Altersetin (**11**) ^b^	[M + H]^+^	400.248	C_24_H_33_NO_4_	400.2479	−0.8
Cyclopenol (**12**) ^b^	[M + H]^+^	311.102	C_17_H_14_N_2_O_4_	311.1023	−1.1
Viridicatol (**13**) ^b^	[M + H]^+^	254.081	C_15_H_11_NO_3_	254.0811	−0.3
Roquefortine C (**14**) ^b^	[M − H]^−^	388.178	C_22_H_23_N_5_O_2_	388.1781	+0.5
Andrastin A (**15**) ^c^	[M + H]^+^	487.269	C_28_H_38_O_7_	487.2689	−3.0

^a^ Values taken from GNPS analysis; ^b^ manually annotated; ^c^ annotated by GNPS; ^d^ data obtained from pure compounds HRMS analysis.

**Table 4:** In vitro activity of compounds **1**, **6**, and **8**.

Compound	MSSA	MRSA	*M. tuberculosis* H37Rv		*M avium* ATCC 15769	Vero Cell ATCC CCL-81 ^6^
			MABA ^4^	LORA ^5^	MABA ^4^	
Alternariol (**1**)			87 (ND)	>50	ND	ND
Viridicatin (**6**)			100 (43.8)	>50	>50	>50
Meleagrin A (**8**)			101 (48.0)	>50	12.3	>50
MIC positive control (μM)	572.41 ^1^	0.86 ^2^	100 (0.03) ^3^	0.08 ^3^	0.05 ^3^	>100 ^3^
**Inhibition level**						
	Total inhibition at 100 μg/mL and partial at 10 μg/mL.					
	Total inhibition at 100 μg/mL.					
	Partial inhibition at 100 μg/mL.					
	No inhibition.					

^1^ Ampicillin; ^2^ Vancomycin; ^3^ Rifampicin; ^4^ % Inhibition at 50 μg/mL (MIC μM); ^5^ MIC μM; ^6^ CC_50_, cytotoxic concentration to 50% inhibition of the cell line. ND, not determined.

## Data Availability

The authors confirm that the data supporting the findings of this study are available within the article and its supplementary material.

## Supplementary Materials

The following are available online, Table S1. Antimicrobial activity of small-scale extracts of marine fungi from the GoM; Table S2. LC-MS data of the 87 fungal extracts used for PCA analysis (features after blank removal); Table S3. Antimicrobial activity of primary fractions of *Alternaria* sp. CIGOM4 and *P. echinulatum* CONTIG4 scaled-up extracts; Table S4. Spectroscopic and spectrometric data of isolated compounds; Table S5. Anti-ESKAPE activity of compounds **1**–**8**; Table S6. Anti-*Mycobacteria* and cytotoxic activities of compounds **1**–**8**; Figure S1. ^1^H NMR spectrum of alternariol (**1**) in methanol-*d_4_* (400 MHz); Figure S2. ^1^H NMR spectrum of methyl alternariol (**2**) in DMSO-*d_6_* (400 MHz); Figure S3. ^1^H NMR spectrum of cyclopenin (**3**) in DMSO-*d_6_* (400 MHz); Figure S4. ^13^C NMR spectrum of cyclopenin (**3**) in DMSO-*d_6_* (100 MHz); Figure S5. ^1^H NMR spectrum of cyclopeptin (**4**) in CDCl_3_ (400 MHz); Figure S6. ^1^H NMR spectrum of dehydrocyclopeptin (**5**) in CDCl_3_ (400 MHz); Figure S7. ^13^C NMR spectrum of dehydrocyclopeptin (**5**) in CDCl_3_ (100 MHz); Figure S8. ^1^H NMR spectrum of viridicatin (**6**) in methanol-*d_4_* (400 MHz); Figure S9. ^13^C NMR spectrum of viridicatin (**6**) in methanol-*d_4_* (100 MHz); Figure S10. ^1^H NMR spectrum of cytochalasin D (**7**) in CDCl_3_ (400 MHz); Figure S11. ^1^H NMR spectrum of meleagrin A (**8**) in CDCl_3_ (400 MHz).

## Sample Availability

Samples of the compounds **1**–**8** are available from the corresponding author.
