# Supplementary material for: Chemical Diversity and Antimicrobial Potential of Cultivable Fungi from Deep-Sea Sediments of the Gulf of Mexico
Source: Molecules. 2021 Dec 2;26(23):7328. doi: 10.3390/molecules26237328 (PMC8659086; doi:10.3390/molecules26237328)
Supplement: Supplementary file 1 [file molecules-26-07328-s001.zip › Supplementary Information_GoM Fungi_rev2.pdf]

SUPPORTING INFORMATION TABLES

Table S1. Antimicrobial activity of small-scale extracts of marine fungi from the GoM.

Strain	Extract Condition	*E. coli* ATCC 10536	*S. typhi* ATCC 9992V	*P. aeruginosa* ATCC 27853	*S. aureus* ATCC 25923	*B. subtilis* ATCC 6633	*C. albicans* ATCC 10231
	A						
P. brevicompactum CONTIG2	B						
	C						
	A						
P. echinulatum CONTIG4	B						
	C						
	A						
Cladosporium sp. CONTIG5	B						
	C						
	A						
C. ramotenellum CONTIG7	B						
	C						
	A						
C. halotolerans CIGOM1	B						
	C						
	A						
Biatriospora sp. CIGOM2	B						
	C						
	A						
S. vesicarium CIGOM3	B						
	C						
	A						
Alternaria sp. CIGOM4	B						
	C						
	A						
Penicillium sp. CIGOM5	B						
	C						
	A						
Biatriospora sp. CIGOM7	B						
	C						
	A						
Penicillium sp. CIGOM8	B						
	C						
	A						
Penicillium sp. CIGOM9	B						
	C						
	A						
Penicillium sp. CIGOM10	B						
	C						
	A						
Penicillium sp. CIGOM11	B						
	C						
	A						
Penicillium sp. CIGOM12	B						
	C						
Penicillium sp. CIGOM13	A						

	B						
	C						
Penicillium sp. CIGOM14	A						
	B						
	C						
Penicillium sp. CIGOM15	A						
	B						
	C						
Penicillium sp. CIGOM16	A						
	B						
	C						
Penicillium sp. CIGOM17	A						
	B						
	C						
Penicillium sp. CIGOM19	A						
	B						
	C						
Penicillium sp. CIGOM20	A						
	B						
	C						
Penicillium sp. CIGOM21	A						
	B						
	C						
Penicillium sp. CIGOM22	A						
	B						
	C						
Penicillium sp. CIGOM23	A						
	B						
	C						
Penicillium sp. CIGOM24	A						
	B						
	C						
Penicillium sp. CIGOM25	A						
	B						
	C						
Penicillium sp. CIGOM26	A						
	B						
	C						
Penicillium sp. CIGOM27	A						
	B						
	C						
MIC positive control (µg/mL)		1.56[1]	0.39[1]	6.25[2]	0.05[1]	16.3[1]	2.5[3]

Inhibition level

	Total inhibition at 200 µg/mL and 20 µg/mL.
	Total inhibition at 200 µg/mL.
	Partial inhibition at 200 µg/mL and 20 µg/mL.
	Partial inhibition at 200 µg/mL.
	No inhibition.

[1] Ampicillin; [2] Gentamicin, [3] Nystatin.

Rice medium at A) RT, light/darkness 12/12 h, 21 d; B) 20 °C, darkness, 30 d; and C) 4 °C, darkness, 60 d.

Table S2. LC-MS data of the 87 fungal extracts used for PCA analysis (features after blank removal).
(Table attached in separate file)

Table S3. Antimicrobial activity of primary fractions of *Alternaria* sp. CIGOM4 and *P. echinulatum* CONTIG4 scaled-up extracts.

	B. subtilis **ATCC 6633**	***S. aureus*** **ATCC 25923**	***C. albicans*** **ATCC 10231**	***S. typhi*** **ATCC 9992V**	***E. coli*** **ATCC 10536**	***P. aeruginosa*** **ATCC 27853**
Extract CIGOM4[a]						
F1						
F2	NT	NT	NT	NT	NT	NT
F3						
F4						
F6						
F7						
F8						
F9						
F10						
Extract CONTIG4[b]						
F1						
F2						
F3						
F4						
F5						
F6						
F7						
F8						
F9						
F10						
F11						
F12						
F13						
MIC positive control (µg/mL)	1.25[1]	0.05[2]	20.0[3]	1.25[1]	5.00[2]	1.25[4]

Inhibition level

Total inhibition at 200 µg/mL and 20 µg/mL.
Total inhibition at 200 µg/mL and partial at 20 µg/mL.
Total inhibition at 200 µg/mL.
Partial inhibition at 200 µg/mL.
No inhibition.

[1] Vancomycin; [2] Ampicillin; [3] Nystatin; [4] Gentamicin.
[a] Rice medium at 4 °C in darkness for 60 d; [a] Rice medium at 20 °C in darkness for 30 d.

Table S4. Spectroscopic and spectrometric data of isolated compounds.

Alternariol (**1**): ^{1}H NMR (methanol-d_4, 400 MHz): δ_H 7.28 (1H, d, *J* = 2.2 Hz, H-6), 6.71 (1H, d, *J* = 2.7 Hz, H-5'), 6.63 (1H, d, *J* = 2.6 Hz, H-3'), 6.38 (1H, d, *J* = 2.1 Hz, H-4), 2.77 (3H, s, 6-Me); HRESIMS *m/z* 257.0452 $[M-H]^-$ (calcd. for $C_{14}H_9O_5$ 257.0445, -1.3 ppm, IHD = 10).
Methyl alternariol (**2**): ^{1}H NMR (DMSO-d_6, 400 MHz): δ_H 11.83 (1H, s, 3-OH), 10.41 (1H, s, 4'-OH), 7.24 (1H, d, *J* = 2.0 Hz, H-6), 6.74 (1H, d, *J* = 2.4 Hz, H-5'), 6.66 (1H, d, *J* = 2.4 Hz, H-3'), 6.63 (1H, d, *J* = 2.1 Hz, H-4), 3.92 (3H, s, 5-OMe), 2.75 (3H, s, 6'-Me); HRESIMS *m/z* 273.0755 $[M+H]^+$ (calcd. for $C_{15}H_{13}O_5$ 273.0758, -0.9 ppm, IHD = 10).
Cyclopenin (**3**): ^{1}H NMR (DMSO-d_6, 400 MHz): δ_H 10.86 (1H, s,1-NH), 7.54 (1H, m, H-8), 7.30 (1H, m, H-16), 7.22 (2H, t, *J* = 7.6 Hz, H-15/H-17), 7.16 (1H, d, *J* = 8.0 Hz, H-9), 7.09 (1H, t, *J* = 7.5 Hz, H-7), 6.91 (1H, dd, *J* = 7.7 Hz, 1.4, H-6), 6.63 (2H, d, *J* = 7.6 Hz, H-14/H-18), 4.02 (1H, s, H-10), 3.07 (3H, s, 4-*N*-Me). ^{13}C NMR (DMSO-d_6, 100 MHz): δ_C 165.9 (C-2), 165.3 (C-5), 135.2 (C-11), 132.4 (C-8), 131.0 (C-9), 130.5 (C-13), 128.7 (C-16), 127.9 (C-14/C-18), 126. 4 (C-12), 126.1 (C-15/C-17), 124-2 (C-7), 121.2 (C-6), 70.1 (C-3), 30.9 (4-*N*-Me); HRESIMS *m/z* 295.1074 $[M+H]^+$ (calcd. for $C_{17}H_{15}N_2O_3$ 295.1077, -1.1 ppm, IHD = 12.0).
Cyclopeptin (**4**): ^{1}H NMR ($CDCl_3$, 400 MHz): δ_H 8.56 (1H, br s, 1-NHb), 8.26 (1H, br s, 1-NHa), 8.11 (1H, d, *J* = 7.7 Hz, H-6b), 7.96 (1H, d, *J* = 7.4 Hz, H-6a), 7.53 (1H, t, *J* = 7.4 Hz, H-8b), 7.46 (1H, t, *J* = 7.3 Hz, H-8a), 7.25 (12H, m, H-7, H-13, H-14, H-15, H-16, H-17, H-18), 7.02 (1H, d, *J* = 7.5 Hz, H-9b), 6.94 (1H, d, *J* = 7.9 Hz, H-9a), 4.35 (1H, m, H-3a), 4.26 (1H, m, H-3b), 3.49 (1H, m, H_a-10b), 3.22 (1H, m, H_a-10a), 3.15 (3H, s, 4-*N*-Mea), 2.91 (3H, s, 4-*N*-Meb), 2.85 (1H, m, H_b-10b), 2.71 (1H, m, H_b-10a). HRESIMS *m/z* 281.1282 $[M+H]^+$ (calcd. for $C_{17}H_{17}N_2O_2$ 281.1285, -0.9 ppm, IHD = 11).
Dehydrocyclopeptin (**5**): ^{1}H NMR ($CDCl_3$, 400 MHz): δ_H 8.22 (1H, s, 1-NH), 8.02 (1H, dd, *J* = 7.9 Hz, 1.5, H-6), 7.47 (1H, td, *J* = 8.0, 8.0, 1.6 Hz, H-8), 7.37 (5H, m, H-14, H-15, H-16, H-17, H-18), 6.99 (1H, dd, *J* = 8.1, 0.9 Hz, H-9), 6.95 (1H, s, H-10), 7.27 (1H, m, H-7), 3.19 (3H, s, 4-*N*-Me). ^{13}C NMR ($CDCl_3$, 100 MHz): δ_C 171.5 (C-2), 166.8 (C-5), 135.5 (C-11), 133.4 (C-13), 132.8 (C-6), 132.1 (C-3), 131.6 (C-7/C-8), 130.0 (C-16), 129.6 (C-14/C-18), 129.1 (C-15, C-17), 125.7 (C-12), 125.3 (C-9), 120.5 (C-10), 36.2 (4-*N*-Me); HRESIMS *m/z* 279.1126 $[M+H]^+$ (calcd. for $C_{17}H_{15}N_2O_2$ 279.1128, -0.7 ppm, IHD = 12).
Viridicatin (**6**): ^{1}H NMR (methanol-d_4, 400 MHz): δ_H 7.52 (2H, t, *J* = 7.3 Hz, H-3'/H-5'), 7.45 (1H, d, *J* = 7.3 Hz, H-8), 7.37 (4H, m, H-6/H-2'/H-4'/H-6'), 7.19 (1H, d, *J* = 8.1 Hz, H-5), 7.11 (1H, m, H-7). ^{13}C NMR (methanol-d_4, 100 MHz): 160.5 (C-2), 143.4 (C-3), 135.1 (C-10), 134.1 (C-1'), 131.2 (C-2'/C-6'), 129.5 (C-3'/C-5'), 129.0 (C-4), 128.0 (C-5), 126.1 (C-4'), 123.9 (C-6'/C-7'), 123.0 (C-9), 116.5 (C-8); HRESIMS *m/z* 238.0859 $[M+H]^+$ (calcd. for $C_{15}H_{12}NO_2$ 238.0863, -1.5 ppm, IHD = 11).
Cytochalasin D (**7**): ^{1}H NMR ($CDCl_3$, 400 MHz): δ_H 7.31 (2H, t, *J* = 7.6 Hz, H-2',H-6'), 7.24 (1H, m, H-4'), 7.13 (2H, d, *J* = 6.9 Hz, H-3',H-5'), 6.11 (1H, dd, *J* = 15.7, 2.7 Hz, H-19), 5.69 (1H, dd, *J* = 15.6, 9.8 Hz, H-21), 5.48 (1H, br s, 2-NH) 5.33 (1H, dt, *J* = 10.0, 5.1 Hz, H-20), 5.30 (1H, br s, H_a-12), 5.14 (1H, dd, *J* = 15.8, 2.3 Hz, H-14), 5.09 (1H, br s, H_b-12), 4.65 (1H, br s, H-13), 3.81 (1H, d, *J* = 10.5 Hz, H-7), 3.23 (1H, dt, *J* = 8.4, 3.7 Hz, H-3), 2.83 (1H, m, H-10), 2.73 (1H, m, H-5), 2.67 (1H, m, H-4), 2.51(1H, dt, *J* = 13.0, 11.0 Hz, H-16), 2.26 (3H, s, H-25), 2.14 (1H, dd, *J* = 4.5, 3.5 Hz, H-8), 2.02 (1H, dd, *J* = 12.9, 5.1 Hz, H-15), 1.51 (3H, s, H-23), 1.19 (3H, d, *J* = 6.8 Hz, H-22), 0.95 (3H, d, *J* = 6.7 Hz, H-11). HRESIMS *m/z* 508.2689 $[M+H]^+$ (calcd. for $C_{30}H_{38}NO_6$ 508.2693, -0.9 ppm, IHD = 13).
Meleagrin A (**8**): ^{1}H NMR ($CDCl_3$, 400 MHz): 12.90 (1H, br s, 14-NH), 8.29 (1H, s, H-15), 7.97 (1H, d , *J* = 7.8 Hz, H-7), 7.73 (1H, s, H-18), 7.57 (1H, d, *J* = 7.5 Hz, H-4), 7.38 (1H, s, H-20), 7.29 (1H, t, *J* = 7.6 Hz, H-6), 7.09 (1H, t, *J* = 7.6 Hz, H-5), 6.80 (1H, br s, 17-NH), 6.13 (1H, br s, H-22), 5.51 (1H, s, H-8), 5.12 (1H, d, *J* = 18.6 Hz, H_a-23), 5.08 d (1H, d, *J* = 13.0 Hz, H_b-23), 3.74 (3H, s, 1-OMe), 1.35 (3H, s, Me-25), 1.25 (3H, s, Me-24). HRESIMS *m/z* 434.1820 $[M+H]^+$ (calcd. for $C_{23}H_{24}N_5O_4$ 434.1823, -0.6 ppm, IHD = 15).

Table S5. Anti-ESKAPE activity of compounds **1-8**.

Compound	MSSA	MRSA	VREF	VSRF	***K. aerogenes*** **ATCC 13048**	***E. cloacae*** **ATCC 700324**	***K. pneumoniae*** **ATCC 700603**	***A. baumannii*** **strain 564**	***A. baumannii*** **ATCC 17978**
Alternariol (**1**)									
Alternariol methyl ether (**2**)									
Cyclopenin (**3**)									
Cyclopeptin (**4**)									
Dehydrocyclopeptin (**5**)									
Viridicatin (**6**)									
Cytochalasin D (**7**)									
Meleagrin A (**8**)									
MIC positive control (μg/mL)	572.41[1]	0.86[2]	4.31[2]	2.59[2]	2.62[3]	2.09[3]	39.26[3]	>7328.31[3]	5.23[3]

**Inhibition level**	
	Total inhibition at 100 μg/mL and partial at 10 μg/mL.
	Total inhibition at 100 μg/mL.
	Partial inhibition at 100 μg/mL.
	No inhibition.

[1] Ampicillin; [2] Vancomycin; [3] Gentamicin.

Table S6. Anti-*Mycobacteria* and cytotoxic activities of compounds **1-8**.

Compound	***M. tuberculosis H37Rv***		***M abscessus*** **ATCC 19977**	***M. chelonae*** **ATCC 35752**	***M. marinum*** **ATCC 927**	***M. avium*** **ATCC 15769**	***M. kansasii*** **ATCC 12478**	**Vero cell ATCC CCL-81[4]**
	**MABA[1]**	**LORA[2]**	**MABA[3]**					
Alternariol (**1**)	87 (ND)	>50	ND	ND	ND	ND	ND	ND
Alternariol methyl ether (**2**)	68 (ND)	ND	ND	ND	ND	ND	ND	ND
Cyclopenin (**3**)	29 (ND)	ND	ND	ND	ND	ND	ND	ND
Cyclopeptin (**4**)	20 (ND)	ND	ND	ND	ND	ND	ND	ND
Dehydrocyclopeptin (**5**)	30 (ND)	ND	ND	ND	ND	ND	ND	ND
Viridicatin (**6**)	100 (43.8)	>50	>50 (0)	>50 (37)	>50 (10)	>50 (21)	>50 (48)	>50
Cytochalasin D (**7**)	29 (ND)	ND	ND	ND	ND	ND	ND	ND
Meleagrin A (**8**)	101 (48.0)	>50	>50 (0)	>50 (18)	>50 (81)	12.25	>50 (19)	>50
MIC Rifampicin (μg/mL)	100 (0.03)	0.08	>8.0	4.25	0.09	0.05	0.41	>8.0

[1] % Inhibition at 50 μg/mL (MIC μM); [2] MIC μM; [3] MIC μM (% Inhibition); [4] CC_{50}, cytotoxic concentration to 50% inhibition of the cell line. ND, not determined

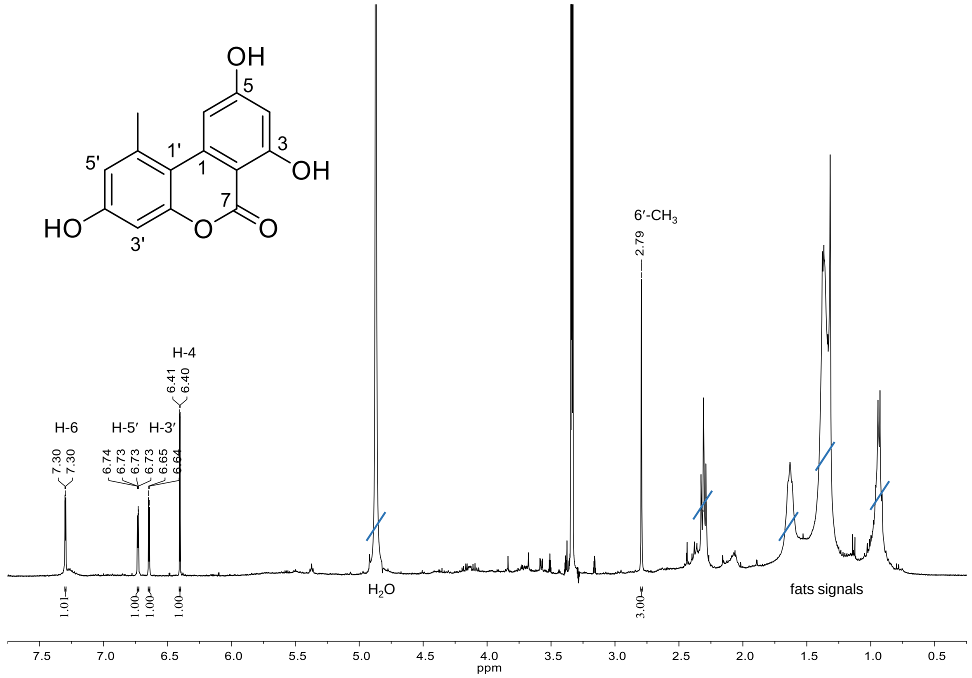

Figure S1. ^{1}H NMR spectrum of alternariol (**1**) in methanol-d_4 (400 MHz).

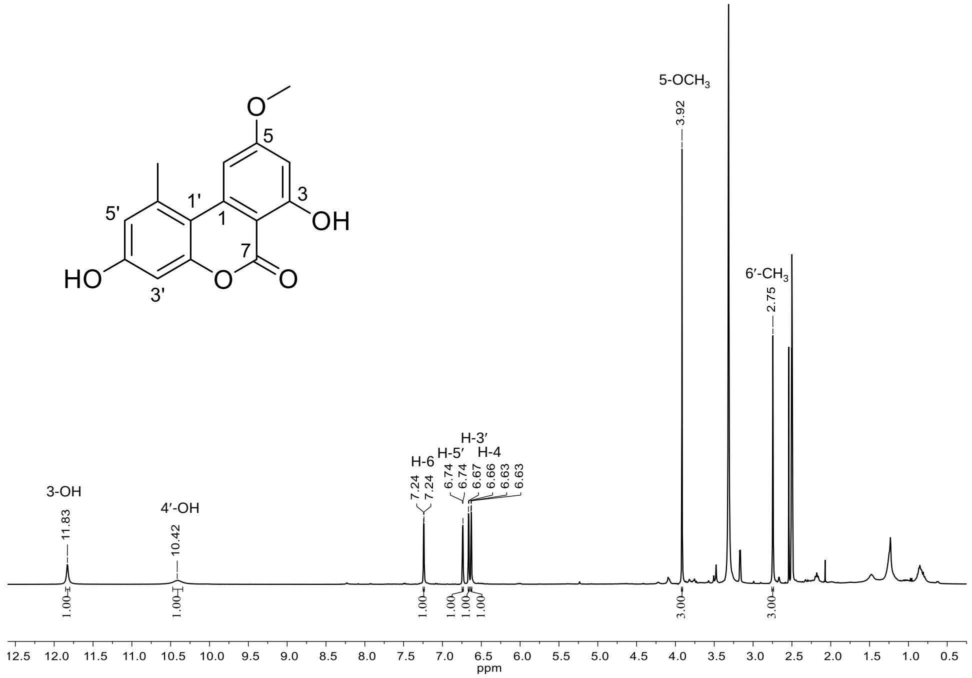

Figure S2. ^{1}H NMR spectrum of methyl alternariol (**2**) in DMSO-d_6 (400 MHz).

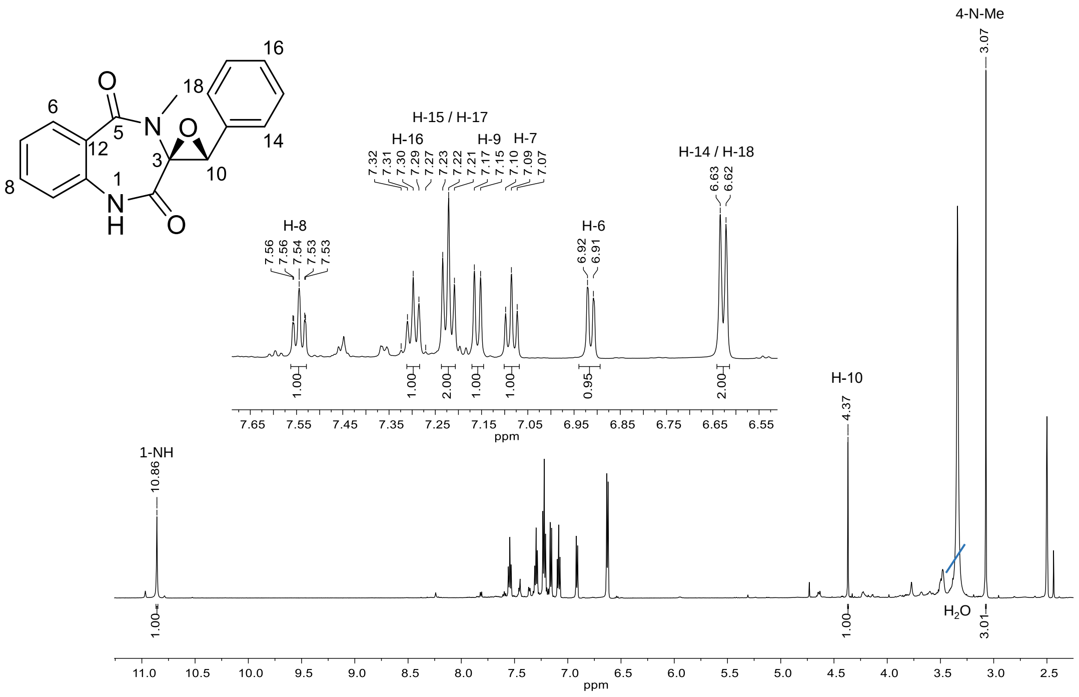

Figure S3. ^{1}H NMR spectrum of cyclopenin (**3**) in DMSO-d_6 (400 MHz).

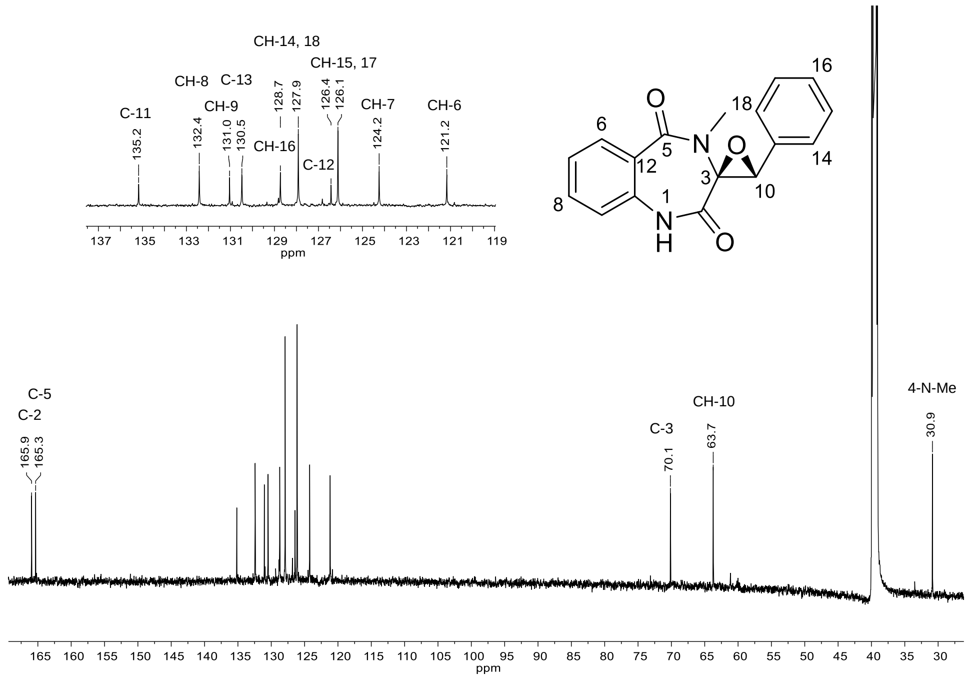

Figure S4. ^{13}C NMR spectrum of cyclopenin (**3**) in DMSO-d_6 (100 MHz).

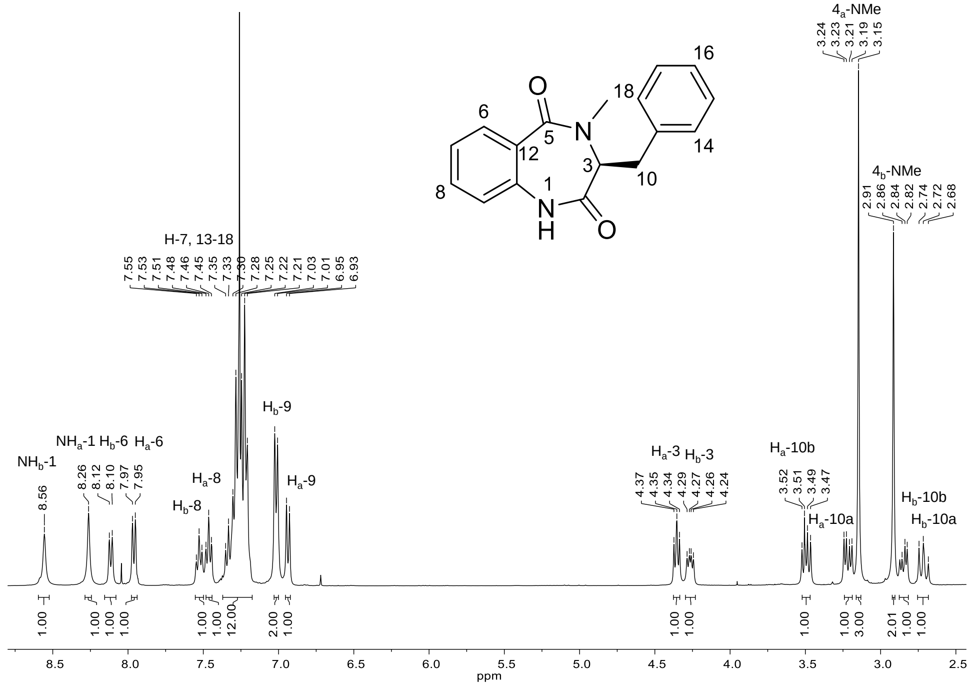

Figure S5. ^{1}H NMR spectrum of cyclopeptin (**4**) in $CDCl_3$ (400 MHz).

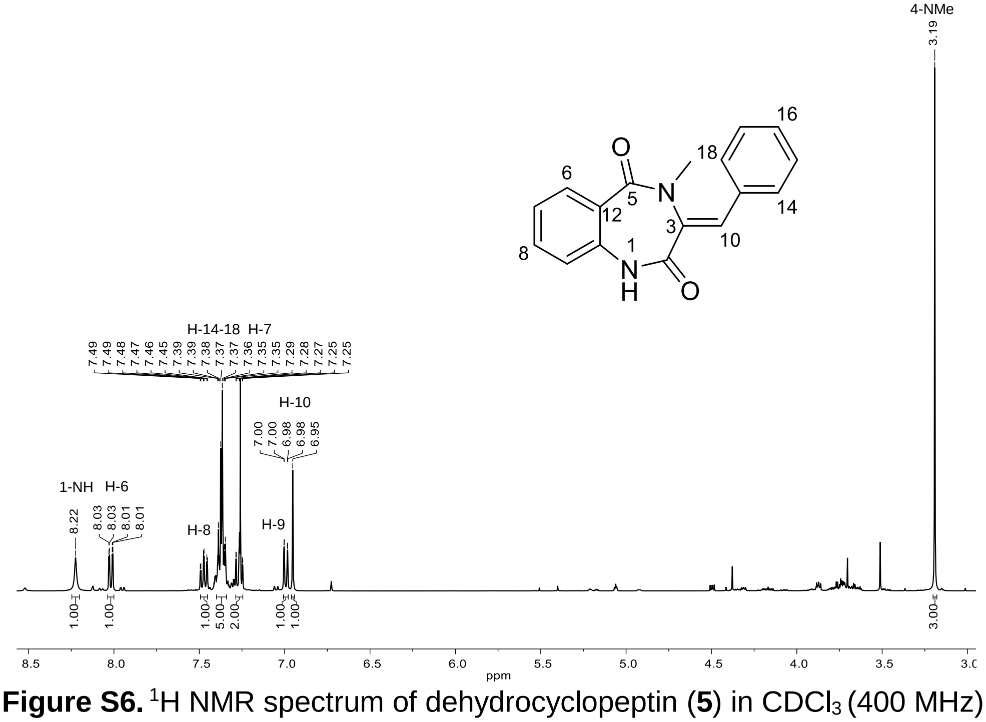

Figure S6. ^{1}H NMR spectrum of dehydrocyclopeptin (**5**) in $CDCl_3$ (400 MHz).

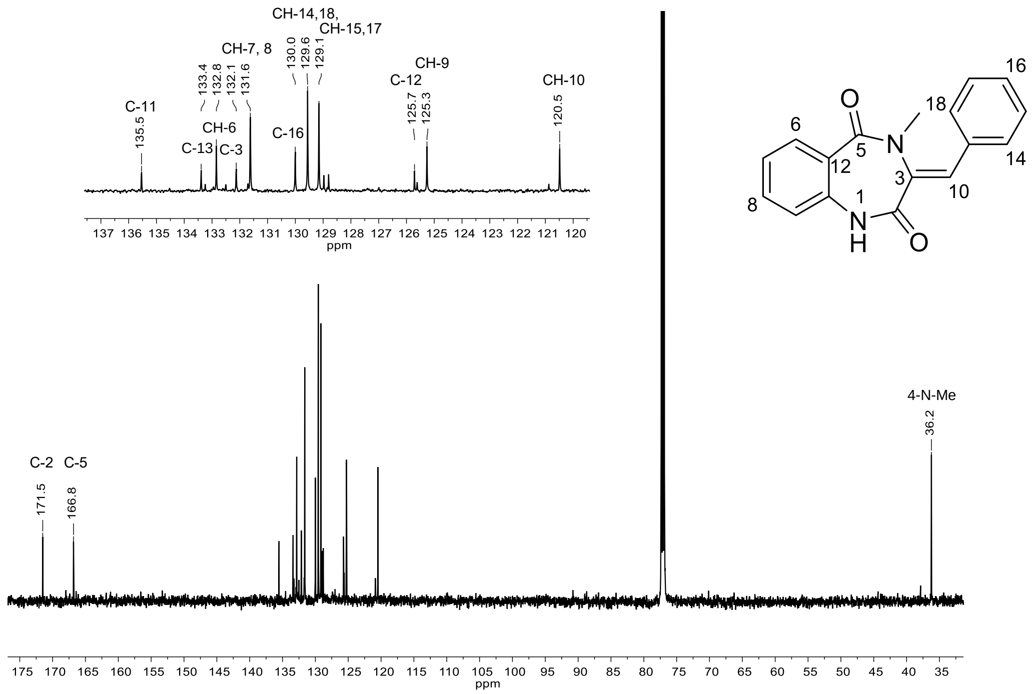

Figure S7. ^{13}C NMR spectrum of dehydrocyclopeptin (**5**) in $CDCl_3$ (100 MHz).

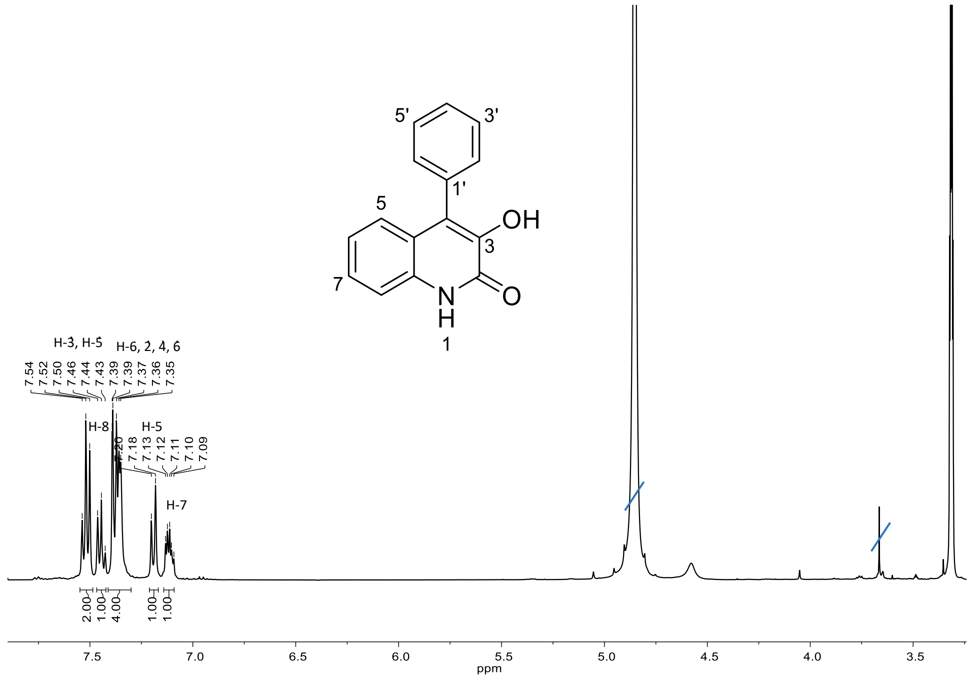

Figure S8. ^{1}H NMR spectrum of viridicatin (**6**) in methanol-d_4 (400 MHz).

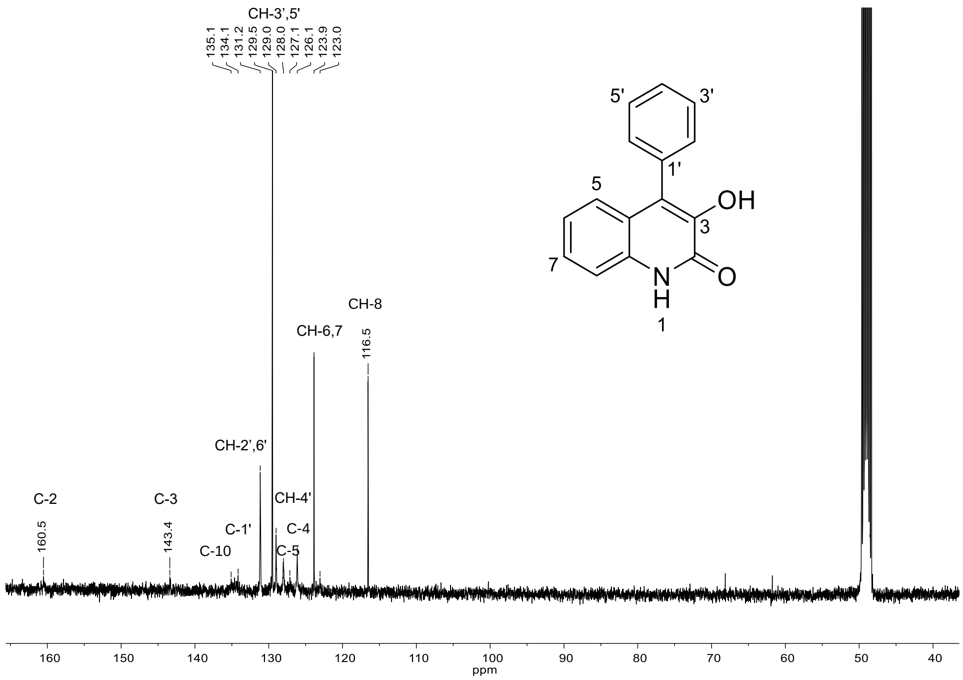

Figure S9. ^{13}C NMR spectrum of viridicatin (**6**) in methanol-d_4 (100 MHz).

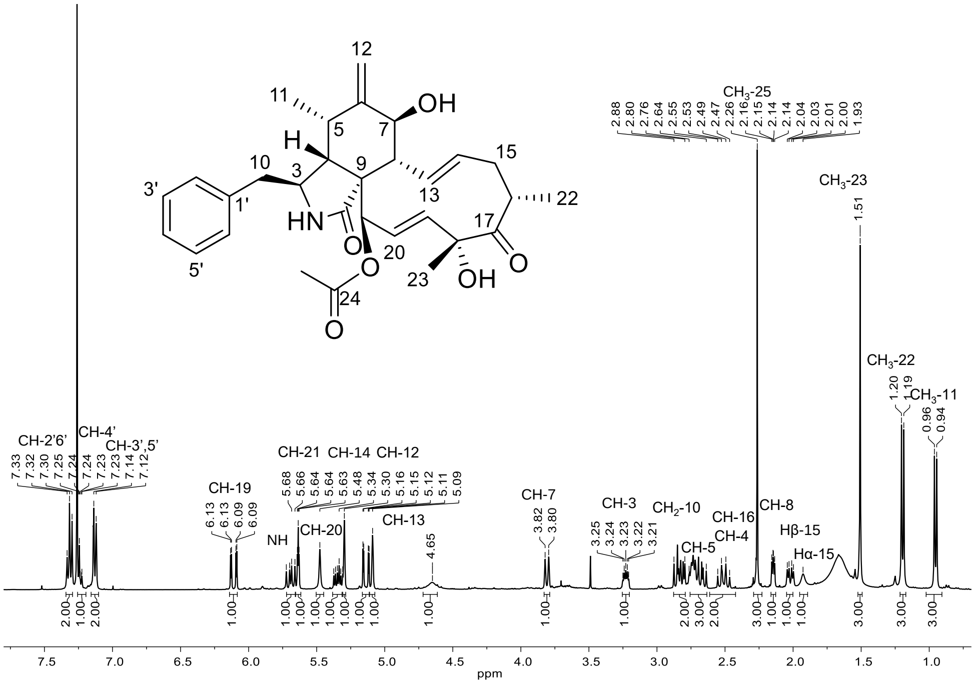

Figure S10. ^{1}H NMR spectrum of cytochalasin D (**7**) in $CDCl_3$ (400 MHz).

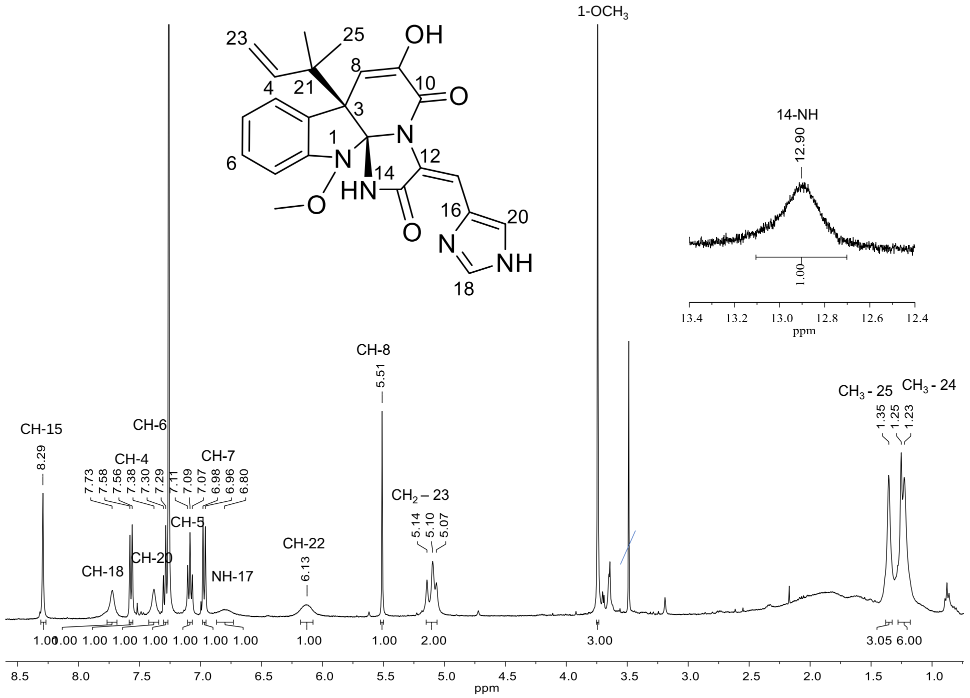

Figure S11. ^{1}H NMR spectrum of meleagrin A (**8**) in $CDCl_3$ (400 MHz).
